# Circular and Green Sustainable Polymer Science

**DOI:** 10.3390/polym17081036

**Published:** 2025-04-11

**Authors:** Antonio Pizzi

**Affiliations:** LERMAB, Laboratoire d’Etude et de Recherche sur le MAteriau Bois, Université de Lorraine, 27 rue Philippe Seguin, CS60036, 88021 Epinal, France; antonio.pizzi@univ-lorraine.fr

The intense search for polymeric materials derived not from oil but from renewable resources, based on novel approaches and aiming to achieve superior performances and lower costs, is evident across the entire field of polymer chemistry today. This Special Issue, dedicated to Circular and Green Polymer Science, aims to collect cutting-edge original research papers and reviews on the main areas where novel approaches—conceptual or applied—are being taken to the fundamental chemistry, mechanisms, applications, engineering, and technologies of “Green” biosourced polymers. With the development of sustainable materials in particularly sharp focus, the contributions to this Special Issue range in topic across the vast variety of polymers in use today, with none being excluded, even including mixed synthetic/biosourced semigreen polymers. An important focus of this SI is on “Circular” Polymer Science and Technology, including the important aspect of recycling, a topic that has acquired particular importance. This notion of “Circular” science particularly means paying attention to “Sustainable Development”, an especially relevant subject which will only become more important in the future. Further considering Polymer Science and Technology for Environmental Ecology, this Special Issue is particularly focused on what is now, and will become moreso in the future, of primary importance in all the sciences, including but not limited to the following research areas:

PLA sustainable polymers and their applications;

All Biosourced Sustainable Polymers and their applications;

The Environmental Sustainability of Green Polymers, and their conception, application, and methodology;

The recycling of polymeric natural and synthetic fibers;

The recycling of natural and synthetic polymers;

Cellulose-derived bioplastics and films;

Eco-friendly polymeric natural and synthetic adhesives;

The biodegradation and recycling of synthetic, biological, and industrial polymers;

Recycled/recyclable sustainable fillers for biosourced and synthetic polymers;

The multi-cycle physical recycling of synthetic and biosourced polymers;

Composites for application in Sustainable Buildings;

Natural fiber reinforcement for the sustainable development of thermoplastics.

